# A Review of Robotic Surgery: Engineering Considerations and Future Directions From Surgeons' Perspectives

**DOI:** 10.1002/rcs.70238

**Published:** 2026-10-06

**Authors:** Noah Shaikh, Walter Fitschen, Abhinaba Bhattacharjee, Dimitrios Stefanidis, Michael W. Sim, Meghan T. Turner, Sohel Anwar, Muhammad Ashraful Alam

**Affiliations:** ^1^ Department of Otolaryngology Indiana University School of Medicine Indianapolis Indiana USA; ^2^ School of Electrical and Computer Engineering Purdue University College of Engineering West Lafayette Indiana USA; ^3^ School of Mechanical Engineering Purdue University College of Engineering West Lafayette Indiana USA; ^4^ Department of Surgery Indiana University School of Medicine Indianapolis Indiana USA; ^5^ Department of Otolaryngology West Virginia University School of Medicine Morgantown West Virginia USA; ^6^ Department of Mechanical Engineering Purdue University Indianapolis Indiana USA

**Keywords:** telerobotics, engineering, haptic technology, invasive surgery, learning curve, medicine, robotic surgery, teleoperation

## Abstract

**Introduction:**

Since its initial inception, robotic‐assisted minimally invasive surgery (RAMIS) is continually being applied to a wider array of procedures. However, there are significant limitations in current surgical subsystems, including a large learning curve, lack of effective haptic feedback, decreased surgical intuition, and decreased dexterity.

**Methods:**

A narrative review of the literature was conducted to provide an overview of key developments in the field. Relevant articles were identified through searches of PubMed, IEEE, Google Scholar, and EMBASE, along with manual screening of references.

**Results:**

A selection of representative articles covering topics including teleoperation, kinematics, dynamics, and haptic feedback is discussed to highlight major themes and recent advances.

**Conclusions:**

Despite recent advances, limitations to operator experience remain. Numerous strategies and approaches are currently being proposed and explored to further advance surgical subsystems, improving dexterity, haptic feedback, movement time delay, and learning curve.

## 1 Introduction

Minimally invasive surgery (MIS) represents a subset of surgical techniques that allow for reduced surgical trauma, risk, scarring, blood loss, infection, pain, and recovery time. MIS can be performed through thin, tubed, retracting channels called trocars, through which miniaturised laparoscopic surgical instruments are passed (Figure 1) [1]. Limitations of dexterity, precision, visualisation, and ergonomic control with MIS have gradually been overcome with advances in robotic assistance. In robotic‐assisted minimally invasive surgery (RAMIS), the laparoscopic tool has a wider workspace, surgeon physiologic tremors can be dampened, and improved ergonomics can reduce surgeon strain [1]. However, the lack of accurate force feedback, bidirectional computer‐robot latency, and cost can limit the safety and availability of these systems. Additionally, there is a steep learning curve for robotic surgery, with certain surgeries, such as aortic valve replacement, requiring years of training and nearly 500 cases to achieve mastery [2]. Several reviews have discussed multiple elements in the robotic surgery hardware and software pipeline [3], detailing historical perspectives [1, 3, 4, 5], increasing clinical uptake [1, 5, 6], novel applications [3, 4], force sensing [3, 7], teleoperation [4, 8], haptic feedback [1, 3, 7, 8], novel instrumentation [1, 3, 7], and incorporation of artificial intelligence [1, 4]. However, these reviews are limited in providing a narrow clinical or engineering design focus without the translational surgeon‐centred focus to guide future engineering design priorities. Therefore, the purpose of this review is two‐fold: 1) to delineate a generalised overview of important surgical robotic subsystems, and 2) to discuss current limitations and open problems for future research with a surgeon‐focused perspective (Figure 2). This review specifically focuses on engineering subsystems of particular clinical relevance to surgeons including teleoperation, kinematics and dynamics, force and tactile sensing, haptic feedback, and surgical intuition. Given the critical need of accurate end effector (i.e., surgical instrument tip) sensing and haptic feedback, there is extra detail focused on these subjects.

**FIGURE 1:**
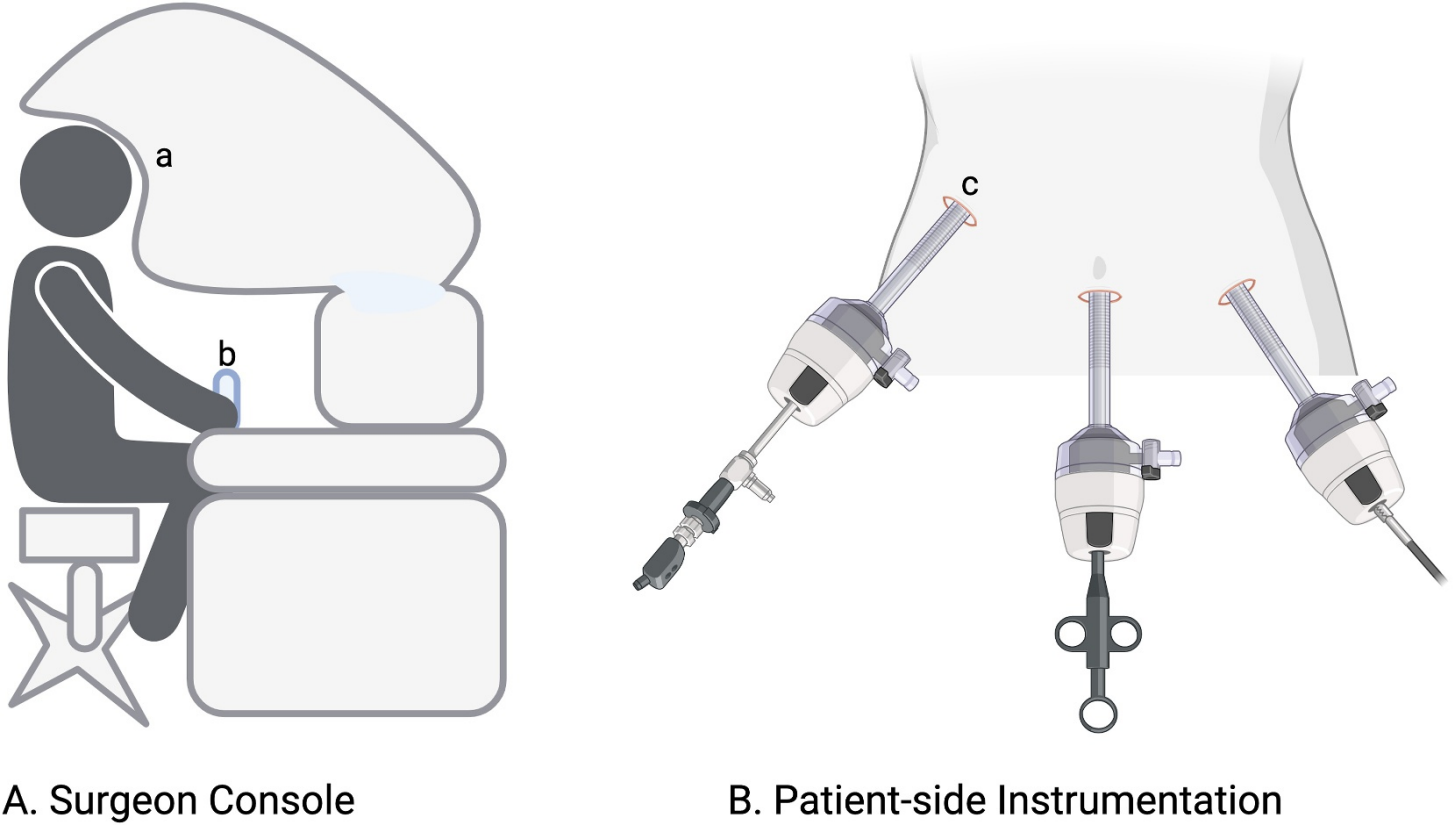
(A) Surgeon‐Side console, which is often designated as a master tool manipulator; a: 3D vision eyepiece b: Surgeon haptic controller. (B) Patient‐side laparoscopic tools can be decoupled from direct surgeon contact through a teleoperated console; c: Incision through patient's body through which a surgical port, trocar, is placed. Surgical instruments are placed through trocar port.

**FIGURE 2:**
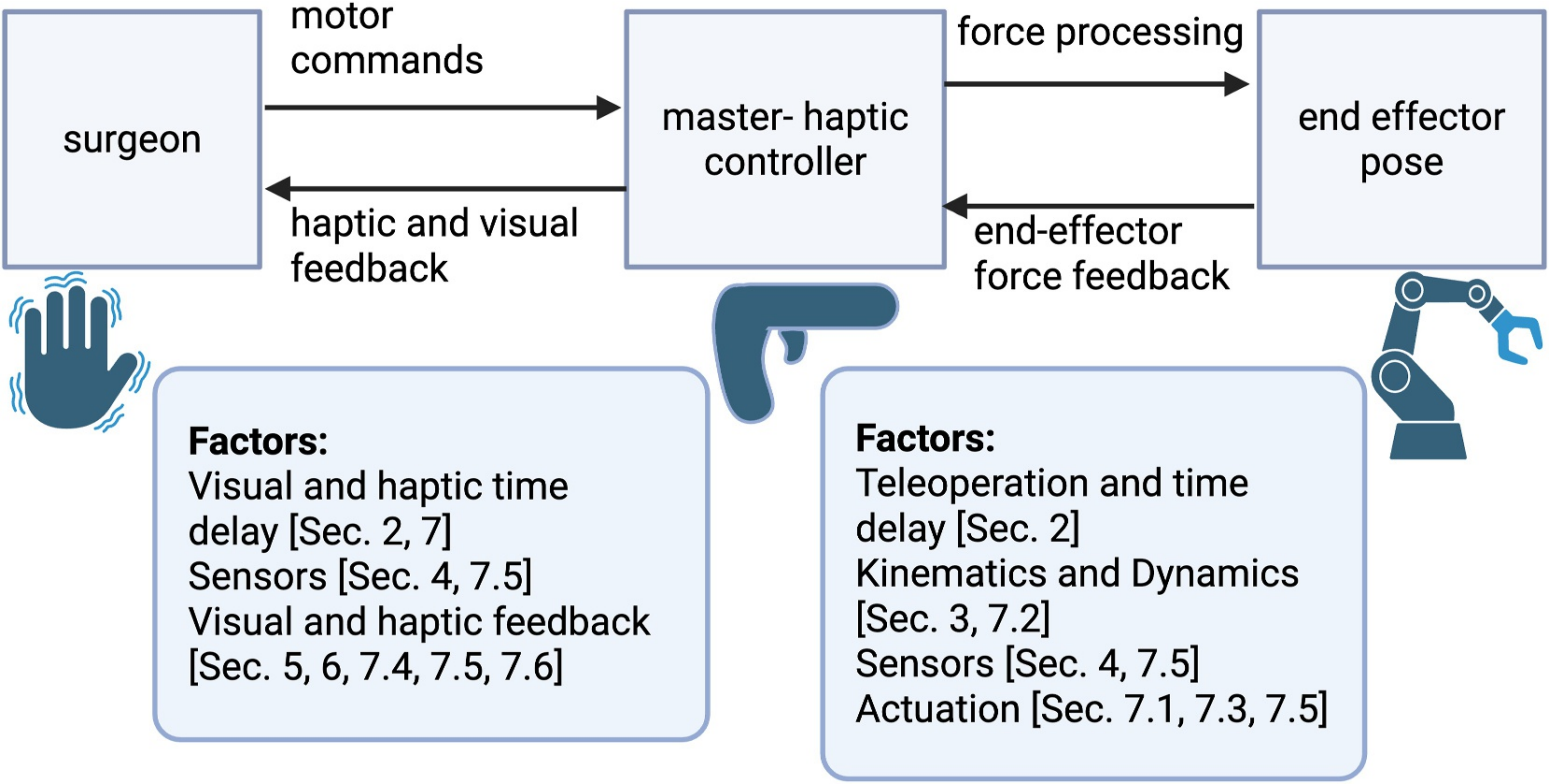
A general scheme of the robotic workflow. A surgeon sends motor commands to a remotely located robot through a haptic controller. This teleoperated surgical robot may be located in the same operating room or even across the world. Kinematics and dynamics algorithms are used to optimise the targeted robot trajectory and end position and orientation. The robot, through its interaction with the tissue and environment, conveys haptic and visual feedback to the operating surgeon who then makes adjustments to correct current pose or plan the next manoeuvre.

## 2 Methods

A narrative review of the current landscape of the literature was conducted. Focus was placed on providing an overview of key developments in the field with an emphasis on current challenges and possible solutions. Searches were conducted on public databases, including PubMed, IEEE, Google Scholar, and EMBASE from the beginning of database inception until July 6, 2026. The initial search was conducted on March 13, 2025, with repeat periodic searches until article submission on July 6, 2026. The search included combinations of the following keywords or phrases: “robotic surgery”, “surgical robot*“, “robot‐assisted minimally invasive surgery”, “RAMIS”, “telesurgery”, “teleoperation”, “kinematics”, “dynamics”, “sensors”, “force sensing”, “haptic sensing”, “autonomous”, “artificial intelligence”, “haptic feedback”, and variations on these terms to incorporate a wide variety of articles. Peer‐reviewed original research articles, review articles, narrative summaries, peer‐reviewed perspective articles, and conference papers were included. Articles were eligible if they included a discussion of robotic surgery history, current robotic surgery instrumentation, teleoperation, kinematics or dynamics, force or tactile sensing, surgical visualisation, surgical ergonomics, surgical intuition, development of novel surgical instruments, challenges in robotic surgery, and non‐robotic surgery technology that could potentially be incorporated into robotic surgery. Non‐peer‐reviewed articles, articles without applicability to robotic surgery, duplicate publications, or articles lacking sufficient technical or clinical detail were excluded. Additional articles were identified through the references in analysed studies. Information extracted included date of publication, study methodology, study results, innovation, and, if not specifically involving a robotic surgery platform, the potential applicability of the technology to robotic surgery. Due to the nature of this work being focused on already published literature, no human or animal subjects were involved, and ethical approval was not required. Because this article was designed as a narrative review rather than a systematic review or meta‐analysis, formal risk‐of‐bias assessment and pooled quantitative analysis were not performed. To reduce selection bias, article selection was guided by inclusion/exclusion criteria and the predefined thematic domains of robotic surgery teleoperation, kinematics and dynamics, force and tactile sensing, and visual and haptic feedback. Articles were selected to provide representative coverage of major subsystem domains and to highlight engineering challenges with direct relevance to surgeon performance, operative safety, and future robotic surgical development.

## 3 Results

### 3.1 Teleoperation

#### 3.1.1 Background

Master‐slave teleoperation, in which a surgeon remotely controls a slave, is frequently used for robotic surgery (Figure 1) [9]. In a typical position‐force architecture, the master position is sent as a command to the slave, and the slave interaction force is relayed back to the master (Figure 2) [9]. Surgeon commands can include instrument position, orientation, velocity, and camera motion [3]. Stability, robust operation regardless of human operator or environment, and transparency, feeling realistic to the human operator, are important elements in teleoperation [10]. An ideal objective is to have accurate communication of position, velocity, and force signals between the master and slave [11]. The teleoperation signals between the master and slave consoles in RAS require either physical data cable or wireless communication channels. Two of the largest RAS systems, DaVinci (Intuitive, Sunnyvale, CA) [12] and Hugo™(Medtronic, Macquarie Park, NSW) [13], both utilise physical cable connections when using the surgeon and robot console in the same operating room, but the DaVinci has been experimentally used for long‐distance telesurgery [14]. The literature suggests that latency of 0–200 milliseconds (ms) is hardly noticeable by surgeons and is considered ideal for telesurgery. However, surgical functionality decreases after 300 ms and a delay greater than 600 ms is considered unacceptable for any surgery with more than low risk or low complexity [15]. Given that even long‐distance teleoperation with communication over fibre optic cable across distances of up to 1000 km result in a delay under 200 ms [16], the short distance cable‐based teleoperation between the surgeon‐side and patient‐side console located in the same operating room results in a negligible delay. This consideration significantly changes with long‐distance wireless communication.

#### 3.1.2 Challenges and Current Research

One of the major challenges that affects accurate bidirectional signal transmission and creates an operational safety hazard is time delay. This can arise from signal distance, signal relaying, multiplexing (combining multiple signals into a single medium), coding, and decoding [9]. Another source is communication delay that affects system dynamics and causes unwanted and unsafe feedback effects, which can be worsened by the nonlinear transition between free space motion and object contact [17]. Even with modern Fifth‐Generation (5G) wireless networks, these communication delays can have a meaningful effect on motor and visual delays over long distances [18]. Creating a realistic experience in robotic surgery requires the system to respond quickly, feel natural to the surgeon, transmit information reliably, and remain stable during use [9, 17]. One important way to maintain stability is through passivity, which means that the robotic system should not generate extra energy on its own [19]. In practical terms, a passive system can store or dissipate energy, but it should not unexpectedly amplify the surgeon's motion or force input. This helps prevent unstable or unsafe behaviour, especially when the robot is interacting with delicate tissue.

Passivity can be checked mathematically by analysing the system's transfer function or by tracking whether the total energy entering the system remains positive [20]. However, maintaining passivity becomes more difficult when there are communication delays between the surgeon‐side controller and the patient‐side robot [17]. These delays can make force feedback feel inaccurate or cause the system to behave unpredictably.

Several control strategies have been proposed to address this problem. One approach uses an energy reservoir‐based regulator, which monitors and manages the energy exchanged between the surgeon‐side and patient‐side systems. This method has been shown to preserve passivity even with communication delays of up to 200 ms [21]. Another approach uses wave‐variable control, which transforms motion and force signals into energy‐based variables before sending them across the communication channel. By accounting for time delay within these transformed signals, the system can remain stable even when communication is not instantaneous [21]. While it is important to note that wireless communication can introduce time delays and the associated system stability challenges, physical power supply and communication cables can intertwine, decrease degrees of freedom, and crowd a limited workspace [22].

#### 3.1.3 Surgeon's Perspective

From a surgeon's standpoint, teleoperation latency can be experienced as a mismatch between hand movement and the accompanying visual and haptic feedback, which may reduce precision and safety during both fine and gross motor tasks. Teleoperation, when optimal, may allow a surgeon to control a bedside robotic console from any corner of the same operating room and even any corner of the world with a seamless visual and haptic experience. Given the freed patient‐side space that would have been occupied by the operating surgeon and a master console, teleoperation facilitates the surgical robot to approach the patient from nearly any angle, allows for the presence of other patient monitoring devices or the placement of a surgical assistant, and improves surgeon ergonomics. A significant delay in visual or haptic feedback created by the latency of teleoperation can create a nuisance in the best instance. In severe cases, clinically meaningful delay could increase the risk of tissue injury or other adverse events. Surgeons may need to compensate for delays by reducing movement speed, particularly around critical structures. 5G Wireless Networks have been increasingly used to demonstrate the experimental feasibility of long‐distance teleoperation. A recent study on surgical phantoms demonstrated a motor command delay of 18 ms and a video delay of 350 ms over a 300 km distance, with the operating surgeon reporting that the 3D vision was inadequate and the teleoperation usability was insufficient for real surgeries [18]. As innovations in visualisation, vision processing, haptic feedback, and artificial intelligence‐guided surgery increases, this will understandably create potentially much larger data streams that would create an even larger burden on long‐distance and even intra‐operative networks. Finally, there is a growing shortage of surgeons [23], which has most impacted rural populations, with an anticipated decline in general surgeon availability of nearly 1% per year [24]. Rural and under‐served communities with surgeon shortages would benefit greatly from the increased access that long‐distance teleoperation could provide. Increasing innovation in this area will be a necessity to facilitate current data streams for long‐distance teleoperation and the anticipated larger data streams for even local teleoperation.

### 3.2 Kinematics and Dynamics

#### 3.2.1 Background

Kinematic and dynamic models seek to accurately characterise joint and end effector static and dynamic positions. A robotic skeleton can be broken down into elements of joints and limbs. The degrees of freedom (DOF) of a joint are the orthogonal positioning variables that determine the state of the joint. Increasing the number of DOF in a robotic skeleton increases the complexity of kinematic and dynamics calculations. Forward kinematics allow determination of pose, velocity and acceleration of the end effector based on specification of joint parameters [25]. For static pose calculations, this can be expressed as x=f(θ) where x is the pose of the end effector and θ is the vector of joint parameters. For end effector velocity forward calculations, this can be expressed as $\dot{x}=J\left(\theta \right)\dot{\theta }$, where $J=\frac{\delta f}{\delta \theta }$ represents the m×n Jacobian matrix of m joint variables and n end effector operational space variables and characterises the incremental changes in end effector pose with changes in joint position. Utilising the Denavit‐Hartenberg (DH) conventions [26], the orientation matrix of each joint is calculated, and subsequent orientation matrices are multiplied in sequence to obtain the end effector position [25]. Inverse kinematics involves the opposite problem, namely, calculating joint parameters based on provision of the end effector pose. This is mathematically expressed as $\theta =f^{-1}\left(x\right)$ for static pose and $\dot{\theta }=J^{-1}\left(\theta \right)\dot{x}$ for velocity. This problem is considerably more challenging and does not admit to single solutions for certain poses. Forward dynamics calculates joint accelerations from specified joint torques. The reverse problem, calculating joint torques based on end effector position, velocity, and acceleration, is tackled by inverse dynamics. An inertia matrix is formulated to translate joint acceleration to forces. The recursive Newton‐Euler, composite rigid body, and articulated body algorithms can be used for inferring system dynamics [27].

#### 3.2.2 Challenges and Current Research

An ultimate desire of surgical trajectory planning is to allow accurate, smooth displacement, velocity, and acceleration of joints during movement [28]. A major challenge in accurate robotic movement involves solving the inverse kinematics (IK) problem. The IK problem may yield a single solution, multiple solutions, or no solutions, and some solutions may result in a singularity [29]. Analytical solutions to the IK problem are faster but are only available for certain robot designs. Numerical methods, including Newton, Jacobian, and heuristic methods, are used frequently but often run into singularities and are computationally expensive. Novel methods to improve calculation efficiency include creating variable step sizes with an iterable Jacobian and the creation and conversion of a constrained optimization problem followed by a solution with simultaneous perturbation stochastic approximation with a norm‐limited update vector (NLSPSA) [30, 31]. Other approaches to solving the IK problem utilise meta‐heuristic approaches, including the genetic algorithm and particle swarm optimization [29]. Another area of investigation involves the placement of external tracking mechanisms, which may allow for better characterisation of end effector pose to supplement calculations based on kinematics [32].

#### 3.2.3 Surgeon's Perspective

Robotic surgery requires satisfying multiple kinematic constraints, including a collision‐free workspace between the surgeon, patient, and surgical robot, decoupling rotational and translational motion of the end‐effector, free rotation of the end‐effector at every surgical workspace configuration, surgeon movement scaling, high output/input displacement ratio, and, if applicable, kinematic constraints at the surgical incision entry point. Since certain robotic surgery procedures involve placing the manipulator shaft through a trocar, displacements around the trocar insertion site must be minimised to prevent harm to the patient. This constraint can be realized through the remote centre of motion (RCM) [33]. The RCM can be maintained through passive, kinematic, and active (mechatronic) point fixation [34] and enforced through mechanisms including isocenters, circular tracking arcs, parallelograms, synchronous belt transmission, spherical linkages, and parallel manipulators [5]. As further research is performed in robot‐assisted or autonomous surgery, accurate kinematic and dynamic calculations are a necessity for highly accurate tool positioning when the difference of micrometres could make a difference in patient outcomes [35].

### 3.3 Force and Tactile Sensors

Given the importance of accurate robotic pose tracking, inclusion sensing, and the expanding field of autonomous guidance, the creation of accurate end effector sensing is crucial. There are not many sensors that have high resolution, sensitivity, reliability, ease of use, compact design, and low cost, which are factors of particular relevance in robotic surgery [36]. A brief overview of the common sensor classes, including strain‐gauge, capacitive, piezoelectric, and optical sensors will be provided.

#### 3.3.1 Strain Gauge Sensors

##### 3.3.1.1 Background

Strain gauge sensors (Figure 3A) are typically placed on elastic beam structures and create a change in resistance with strain caused by load [37]. The typical bonded metallic strain gauge is a thin metal with electrically insulated backing that is bonded to a surface with adhesive. The gauge is most sensitive to deformation in the major axis and less sensitive to deformation perpendicular to this axis [37]. For multi‐axial detection, individual strain gauges should be mounted with the principal axis in the direction of the measured strain. The resistance of the wire increases with tension and decreases with compression. Variants of a Wheatstone bridge (Figure 4) can be utilised to calculate changes in resistance by measurements through a voltmeter. The equations of relevance have been supplied below, where the gauge factor (GF) approximation is valid at fixed temperature.

**Table:** 

Strain gauge equations	Symbol descriptions	
$\sigma =\frac{F}{A}$	σ	Stress
$\varepsilon =\frac{\Delta L}{L}=\frac{\sigma }{E}$	F	Axial force
$R=\frac{\rho L}{A}$	A	Wire cross sectional area
$\mu =-\frac{\Delta D/D}{\Delta L/L}$	E	Gauge modulus of elasticity
$GF=1+\frac{\Delta \rho /\rho }{\varepsilon }-\frac{\Delta A/A}{\varepsilon }$	D	Wire diameter
$=1+2\mu +\frac{1}{\varepsilon }\frac{d\rho }{\rho }$	R	Resistance of conductor wire
≈1+2μ	L	Conductor wire length
ΔR=R∗ε∗GF	ρ	Material resistivity
$V_{m}=V_{s}\frac{\left(R_{1}+\Delta R_{1}\right)R_{4}-R_{2}R_{3}}{\left(R_{1}+\Delta R_{1}+R_{2}\right)*\left(R_{3}+R_{4}\right)}$	μ	Poisson's ratio
	GF	Gauge factor
	$V_{s}$	Supplied voltage to bridge
	$V_{m}$	Measured voltage across bridge

**FIGURE 3:**
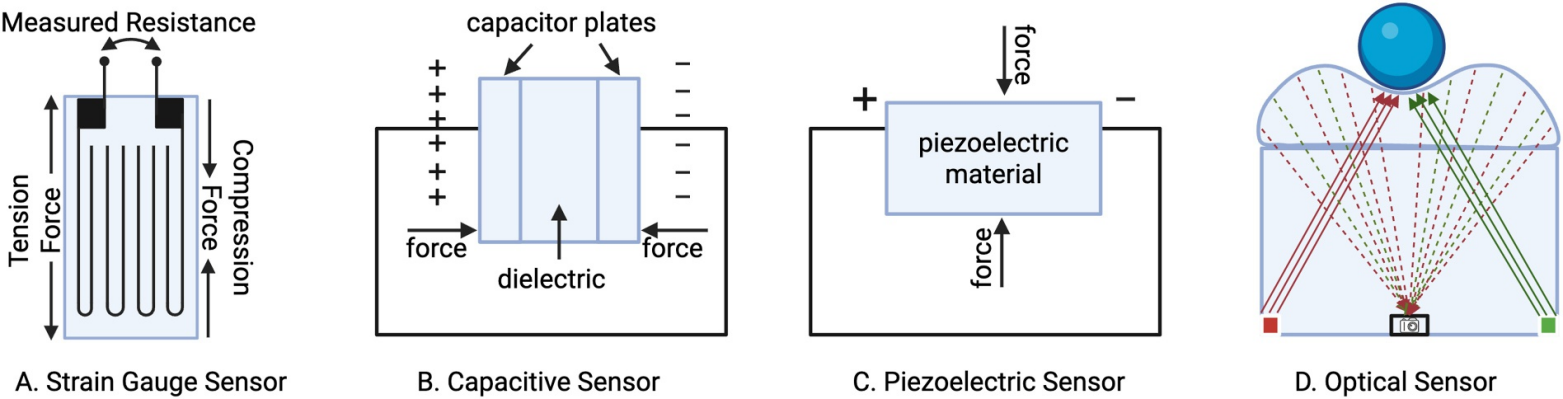
(A) Strain gauge sensor. (B) Capacitive sensor. (C) Piezoelectric sensor. (D) Optical sensor.

**FIGURE 4:**
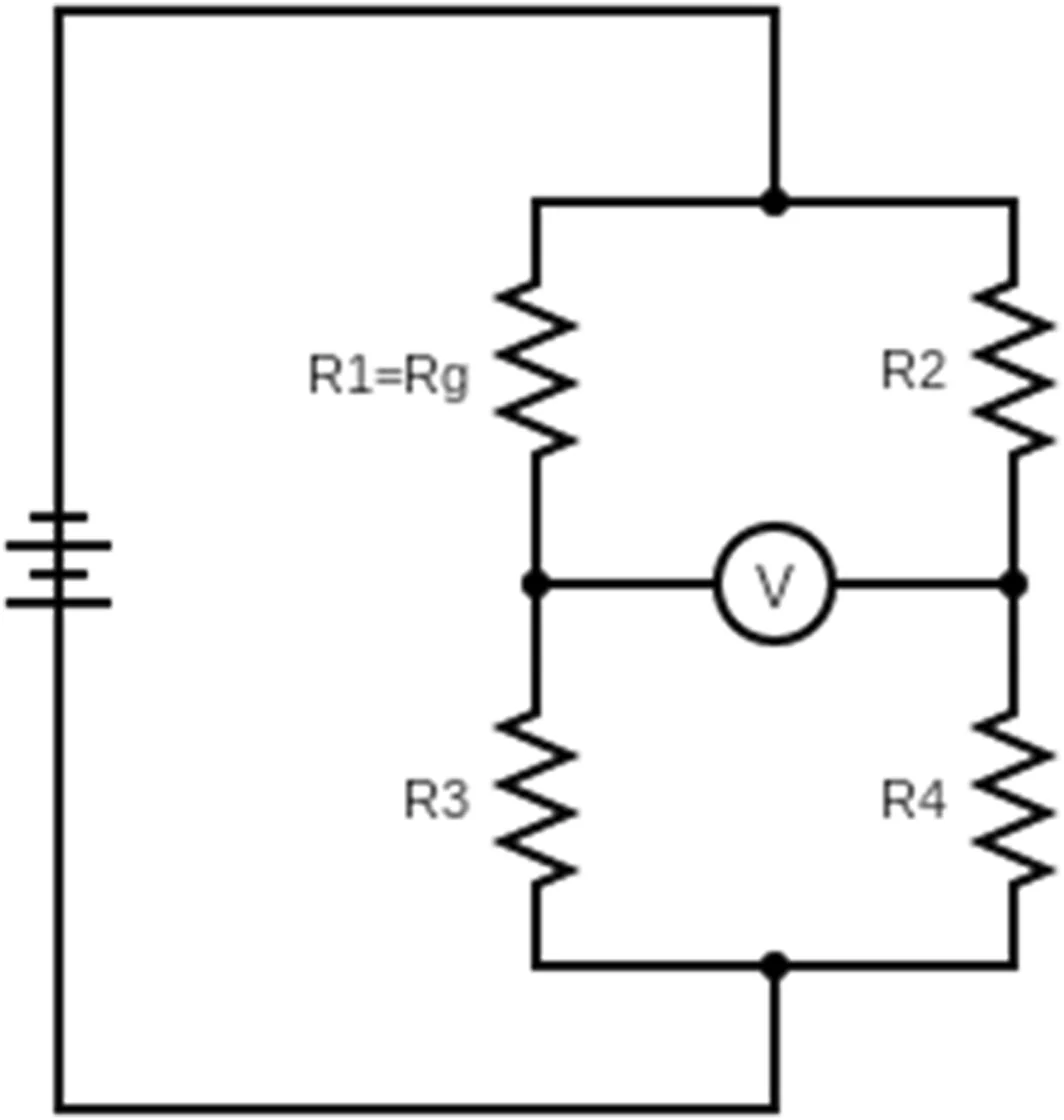
Quarter Bridge Wheatstone bridge. $V_{m}$ is measured voltage across bridge. $V_{s}$ is the supplied voltage by the battery. The strain gauge may be located at any location and number of the resistor positions depending on strain gauge designation, that is, quarter, half, full‐bridge.

Based on the $V_{m}$, forces can be indirectly calculated. As can be seen in the above equations, a ΔR is directly proportional to both the strain, ε and the GF, and, thus, a larger gauge factor can allow for higher strain gauge sensitivity. A balanced bridge condition, producing $V_{m}=0$, occurs when $R_{1}R_{4}=R_{3}R_{4}$. This state can be achieved by setting all resistors equal, $R_{g}=R_{1}=R_{2}=R_{3}=R_{4}$. If utilising four strain gauges in Wheatstone bridge in a full bridge configuration, the partial variations in the measured voltage, $V_{m}$ may be summed giving $\Delta V_{M}=\sum_{i=1}^{4}\frac{\delta V_{M}}{\delta R_{i}}\Delta R_{i}\approx V_{s}*\frac{GF}{4}\left(\varepsilon_{1}-\varepsilon_{2}+\varepsilon_{4}-\varepsilon_{3}\right)$. The right hand, simplified approximation can be made if identical strain gauges are used [37].

##### 3.3.1.2 Challenges and Current Research

Strain gauges are known to be affected by temperature. However, temperature can be compensated for by allowing an adjacent strain gauge to be attached to the identical material and subject to the same temperature variations but shielded from the mechanical strain on the “primary” strained gauge. The subtraction affects, as detailed in the partial variations summations equation, allow for temperature compensation. While half‐bridge and full‐bridge circuits may offer higher sensitivity, quarter‐bridge circuits are more utilised due to simplicity. Semiconductor strain gauges are a material variation in design that allows high resolution and even a negative gauge factor, but these are limited by significantly more temperature sensitivity [37]. Advanced techniques, including geometric changes, disconnection, crack propagation, tunnelling, percolation threshold techniques can be utilised to alter and improve strain gauge sensor resistivity [38].

#### 3.3.2 Capacitive Sensors

##### 3.3.2.1 Background

Capacitive sensors (Figure 3B), classically composed of two parallel electrode layers with interposed dielectric material, can also be used [37]. For a parallel plate capacitor, capacitance is calculated as $C=\frac{\varepsilon_{0}\varepsilon_{r}A}{d}$, where C is capacitance, $\varepsilon_{0}$ is the free‐space dielectric permittivity, $\varepsilon_{r}$ is the relative permittivity of dielectric material, A is the overlapping area of capacitor electrodes, d is the distance between electrodes. The voltage across the capacitor, $V_{M}$, calculates to $V_{M}=\frac{q}{C}$ where q is the charge on the capacitor and C is capacitance. These principles allow force measurement by calibrating change in voltage to change in capacitance which can be correlated to the applied force [37, 39].

##### 3.3.2.2 Challenges and Current Research

The typical parallel capacitor design is optimised for detection of normal force. Shear force sensing by measurement of the overlying capacitor plates has less than a tenth of the normal force sensing discrimination when using parallel plates. However, utilising orthogonal electrodes can improve shear sensitivity by five‐fold [39]. Capacitive sensors have been in less use due to traditional design gauge factor limitations close to 1. However, meticulous geometric and nanoscale material property manipulation innovations have allowed increasing the capacitive gauge factor up to 13. These sensors also have the advantage of low temperature sensitivity [40].

#### 3.3.3 Piezoelectric and Piezoresistive Sensors

##### 3.3.3.1 Background

The direct piezoelectric effect is based on charge generation caused by deformation within a crystal lattice (Figure 3C), and the converse piezoelectric effect generates a force with an applied electric potential. Electrodes mounted on crystal surface can capture the charge generated by the direct piezoelectric effect, which is converted into voltage [37, 41, 42]. The direct piezoelectric effect can be described in very simple form as ΔP=d∗σ, and the converse piezoelectric effect as $x=\frac{\Delta l}{l}=d*E$, where ΔP is projection of change in polarization vector to surface normal, σ is the surface stress, d is the direct/indirect piezoelectric coefficient, x is the relative surface displacement, l is the plate thickness, and E is the projection of the electric field vector to the surface normal. Remarkably, it can be shown that the direct and converse piezoelectric coefficients are equivalent [41, 42]. The capacitance of a piezoelectric material can be described by $C=\frac{\kappa \varepsilon_{0}A}{t}$, where κ is the piezo dielectric constant, A is the electrode area, and t is the crystal thickness. An applied force, F, will induce a charge Q=δ∗F where δ is the piezo charge sensitivity constant. This charge accumulation can be measured by the crystal surface‐mounted electrodes as $V_{0}=\frac{Q}{C}=\frac{\delta *F*t}{\kappa \epsilon_{0}A}$ [37]. Quartz, lead zirconate titanate, ${\text{BaTiO}}_{3}$, polyvinylidene fluoride (PVDF), are frequently studied piezoelectric materials [43]. The most commonly used commercial piezoelectric materials possess ferroelectric properties in which the material can exist in two switchable spontaneous states of polarization that have equal energy and can be alternated with a strong electric field. The process of poling, via heating above the Curie temperature to eliminate spontaneous polarization and then applying an electric field during the cooling process, allows for the creation of a global polarization in even a previously isotropic, non‐piezoelectric material [41, 42]. Piezoelectric sensors, based on the direct piezoelectric effect, have high sensitivity, wide bandwidth (fast and slow changes) and fast response times. Piezoelectric actuators work on the basis of the inverse piezoelectric effect, whereby the application of an electric field causes deformation. These actuators have high resolution, fast response time and capability to produce large forces.

##### 3.3.3.2 Challenges and Current Research

Piezoelectric sensors are frequently limited by fragility, temperature sensitivity, lack of sensitivity to static loads, difficulty in isolating the correct crystal subtype, toxicity, and unwanted triboelectric contact effects [43, 44, 45]. In spite of these limitations, piezoelectric sensors have been utilised in a wide array of novel designs, including complex geometry bio‐mimetic electronic tissue surfaces [46, 47]. Piezoresistive and piezoelectric sensors have been used to simulate fingertip skin for discrete pressure classification, slippage detection and to detect fine structural features, such as braille [47, 48]. They can also be incorporated into flexible dual modal sensors to take advantage of both electromagnetic induction and piezoelectric effects to classify non‐contact approach distance and contact pressure [49]. Given the inherent fragility of ferroelectric crystals, novel designs involving creation of polymer matrix composites can improve flexibility and resilience to stress, water, chemical and thermal damage [50].

#### 3.3.4 Vision Tactile Sensors

##### 3.3.4.1 Background

Vision based tactile sensors are commonly made with a soft elastomer which deforms with contact, an internal camera to detect deformation, and a light‐emitting diode (LED) between elastomer and camera (Figure 3D) [51]. Several variations have been created based on the operating principles of the GelSight sensor [51]. The GelSight sensor surface elastomer, approximated as a neo‐Hookean solid, is made of a clear silicone which may have a matte (fine aluminium) or semi‐specular (bronze or aluminium flakes) coating followed by a diluted fluid silicone surface protective layer [51]. A contacting surface will generate a field motion pattern of the surface markers or surface coating, and the motion magnitude is suggested to be linearly proportional to the force and torque [52]. Marker detection and movement processing can be performed with blob detection, foresight sorting algorithm, and saliency point clouds [53]. Normal force, for example, can be estimated by creating a lookup table with known normal forces and the corresponding pixel intensities [52]. Slip is suspected when the peripheral contact area movement is less than central or shear marker movement. Slip is easiest to detect with flat or small curvature objects which allow for maximum surface area and tracked marker contact [51]. The original GelSight camera had a red, green, and blue filter illuminating the elastomer surface at 60‐degree angles and a sensor range of 60×40 mm, which was only a fraction of the elastomer surface. The use of three LED's allows three measurements per pixel, which over‐constrains the surface normal reconstruction problem and allows easier estimation of two gradient values [54]. This can develop a spatial resolution of 20–30 microns [51]. The original GelSight design has been augmented with innovations in light pattern transmission with the use of light guides, prisms, mirrors, and different LED angles and arrangements [52, 54]. A few variations of the original GelSight image detection design include the OmniTact and the GelStereo sensors. The OmniTact sensor utilises multiple cameras that are directly encased within the gel elastomer [53], and the GelStereo sensor uses two internal cameras with binocular stereo matching algorithm [55]. Several other methods exist for optical force sensing, including the use of Fibre Bragg Gratings and photosensor interruptions [37, 56, 57, 58]. The Fibre Bragg Grating process involves creating a spatially periodic variation in the refractive index of the core of an optical fibre with UV light treatment. A sweeping laser interrogator is used to input a wide spectrum of wavelengths that are reflected back to the interrogator. Input light will only be reflected if it meets Bragg's condition: $\lambda_{B}=2\Lambda n_{\mathrm{eff}}$ where Λ is distance between two grating planes, $n_{\mathrm{eff}}$ is the core fibre refractive index, and $\lambda_{B}$ is the Bragg wavelength [37]. Due to the photoelastic effect, the resultant strain from a force causes a change in the effective refractive index and spacing between the gratings, which ultimately changes the Bragg wavelength [57, 59]. These sensors have been incorporated, for example, into the tip of a laparoscopic grasper to provide real time force feedback [60]. These sensors do have the benefit of small size and resistance to electromagnetic interference. They also have the advantage of being resistant to electromagnetic interference [37].

##### 3.3.4.2 Challenges and Current Research

Optical tactile sensors suffer from multiple limitations in material resilience, light transmission and sensing, and data processing. Mechanical weakness of adhesion between the sensing and support layer and wave guide impurities and edge cracking can affect light transmission. Additionally, the light intensity distribution across the sensing gel is subject to light source condition, optical path consistency and paint homogeneity. The image stream can be affected by gel compression, hardware deformation, and camera shutter speed fluctuation [61]. Additionally, the raw image output is affected by non‐uniform illumination, perspective distortion, and sensor image stream changes. Additional challenges are the wear by frequent grasps, which can cause non‐uniform gel wear and dead pixels and changes in optical path, brightness, and signal strength. Filters and transformation matrices can be used to make output images more uniform for image processing, and non‐uniform gel wear effects can be counteracted with intermittent calibration and dead pixel interpolation [61]. Force detection based solely on point displacement of the 2D elastomer surface can give unrealistic predictions [52]. This can be refined with a numerical approach with the inverse finite element method (iFEM) [52]. Softer gels can improve resolution but have a shorter life span, which can be lengthened with textured protective fabric overlying the sensing surface [61]. A large amount of data is processed in optical sensors. Given the large amount of depth information in every frame, a contact semantic loss function has been used to reduce the amount of data that needs to be processed and improved speed and accuracy of depth information [55]. If ultra high frame rate and long collection time periods are necessary, complementary metal‐oxide‐semiconductor (CMOS) and charge‐coupled device (CCD) cameras can create excess data volume, creating a challenge with real time data processing. This has prompted exploration with event cameras [62]. However, event cameras present challenges with processing slower movements or static deformations.

#### 3.3.5 Sensor Location

The previous overview has discussed general sensor classes that can be incorporated into end‐effector force sensing and the limitations involved. However, sensor placement is also of utmost importance. In order to provide accurate feedback, current sensing mechanisms need to be located within the shaft of the surgical instrument distal to the trocar or within the tip of the instrument itself [3]. In robotic surgery system integrated sensors, the force feedback approach most often involves placement of sensor in the end effector tip [40]. However, there are multiple complexities with embedding sensors in the end effector tip, including issues of size, sterilisation, biocompatibility, fluid resistance [3], wiring [63], tool contamination [64], and dexterity [65]. To combat the issue of sterilisation, a disposable shaft with integrated force sensors, such as strain gauges, has been effective [66]. However, transluminal surgery requires instrument placement through an insertion port; and sensor placement at the shaft is complicated by friction and reaction forces [40]. Previous attempts at alternative site placement have also included incorporating an integrated strain gauge force sensor within the joint motor [67].

#### 3.3.6 Surgeon's Perspective

Novel force and tactile sensor designs and robotic integrations are necessities to facilitate more immersive haptic feedback. Only the most recent models of the DaVinci (Intuitive, Sunnyvale, CA) and Hugo™(Medtronic, Macquarie Park, NSW) incorporate force sensing. The finger of a surgeon has the ability to detect temperature, moisture, normal and shear forces, stretch, slip, tissue compliance, and tissue texture. The accurate quantification and transformation of these forces would allow for more intuitive surgical feedback and would facilitate safer surgery. Currently, there are novel, hand‐held tool sensing mechanisms that have been implemented to assist in normal and abnormal structure identification intraoperatively. Chemical fluorescence with indocyanine green (ICG), which binds plasma proteins, is a well‐established technique for assessing tissue flap vascularity, identifying lymphatic vessels and lymph nodes, and identifying tumours across multiple organ systems [68]. Near‐infrared autofluorescence (NIREF) has been used in thyroid and parathyroid surgery to help properly identify and save at‐risk parathyroid glands to prevent the risk of post‐surgical hypoparathyroidism [69]. In neurosurgery, small electrical impulses can be used to perform intraoperative motor mapping to identify critical brain pathways at risk during tumour resection. This allows maximal safe resection while preserving patient motor function [70]. Additional robotic tools that add these currently available hand‐held tools' functionalities can further amplify the robot's efficacy.

### 3.4 Haptic Feedback

#### 3.4.1 Background

Haptic feedback involves providing the operating surgeon with useful sensory information to inform surgical decision‐making. This can be subdivided into force feedback and tactile feedback. Although force feedback typically focuses on low‐frequency forces sensed by the muscle, joint, and tendon anatomic receptors of the human operator, tactile feedback gives surface texture, roughness, vibration, and skin stretch through skin mechanoreceptor stimulation [8, 71]. To create accurate haptic feedback for a human provider and for considerations of bio‐mimetic sensor design, it is crucial to understand the underlying pathophysiology of the human skin, muscle, and joint sensors (Table 1). Kinesthetic perceptions are related to body position, movement, as well as muscle, tendon and joint forces [74]. The human body provides this perception through proprioception. Tactile sensations come from external stimuli that contact the skin. The important subdivision of tactile sensation arises from the system of mechanoreceptors that detects vibration, light touch, and pressure, and the system of free nerve endings that detects temperature changes and pain. Skin receptors have different minimum stimulus detection thresholds (activation threshold) and different response adjustment speeds to stimuli of various durations. The mechanoreceptor types include Meissner's corpuscles (low frequency vibration, stroking and fluttering movements), Merkel cells (pressure, texture, low frequency vibration), and Ruffini endings (stretch, low frequency vibration), Pacinian corpuscles (high frequency vibrations) [74]. Thermoreceptor nerve endings can perceive a temperature range of 5–45 degrees Celsius [74]. Human skin can perceive force within the range of 100 Pa to 100 kPa [75].

**TABLE 1:** Human mechanoreceptor location, stimulus, and temporal resolution.

Receptor type	Location	Stimulus	Temporal resolution	References
Meissner corpuscle	Dermal papillae	Dynamic deformation	1–300 Hz (most sensitive: 5–50 Hz)	[72, 73]
Ruffini corpuscle	Dermis	Skin stretch, static and dynamic skin indentation	—	[72, 73]
Pacinian corpuscle	Deep dermis	Vibration	5–800 Hz (most sensitive: 30–500 Hz)	[72, 73]
Merkel cell	Epidermis	Static and dynamic indentation	Up to 100 Hz (most sensitive: 0.3–5 Hz)	[72, 73]
Free nerve ending	Epidermis > dermis	Painful force	—	[72, 73]

#### 3.4.2 Challenges and Current Research

As expected, a crucial component in creating accurate feedback involves accurate sensing of the end effector force, which has been previously discussed. After an end effector force has been accurately detected, the next step in the haptic feedback loop is creating a timely, proportionate, non‐disruptive surgeon side force. This can be conveyed with vibrotactile, thermal, pneumatic, hydraulic, shape, actuator‐based memory, and electrotactile systems [76]. Vibrotactile systems create a vibrating motion with reciprocating force, which can be created by direct current (DC) motors, linear resonant actuators (LRA), eccentric rotating mass (ERM), voice coil actuators, or piezoelectric devices. Through the cyclic activation of an electromagnet, an LRA forces a mass to periodically vibrate with a specific frequency. A voice coil creates movement by switching current in a coil positioned within a magnet. An ERM actuator spins an asymmetric mass, creating an asymmetric centripetal force with rotation, and is effective in creating low‐frequency vibrations that allow for a slower response time. Piezoelectric actuators expand when a voltage is applied and have the benefit of small size, fast responses, low cost, and power use. However, they have the disadvantage of requiring high voltage [76, 77]. Thermal feedback is achieved with thermoresistive heaters or Peltier elements. Peltier elements function based on the thermoelectric effect from heat absorption or release through the controlled flow of electric charge between an N‐ and P‐type semiconductors with external current modification [78, 79]. Stiffness perception can also be provided via an external glove exoskeleton, although this is not typical of the design of standard surgical master tool manipulators [80].

#### 3.4.3 Surgeon's Perspective

A lack of effective haptic feedback is the most cited concern about modern surgical robotic systems. The inability to feel what the surgical end effector is in contact with can significantly affect surgical safety, operating time due to slower movements or roundabout approaches to pathology, surgeon trust of the machine, and mounting the robotic surgery learning curve. As sensor technology is able to more accurately quantify the various elements of sensation that a human finger can acquire from the environment, the next frontier is in the safe, timely translation of those sensations into an intuitive format that does not disrupt live movements. Another challenge will be the modification of the surgeon‐side console haptic tool into a wider surface area device such as an exoskeleton glove that allows for better delivery and processing of haptic feedback.

### 3.5 Augmenting Surgical Intuition

#### 3.5.1 Background

The learning curve for robot‐assisted surgery, which naturally depends on the complexity of the procedure, has been described to range from 6 cases for rotator cuff surgery to 500 cases for coronary artery bypass surgery [2, 6]. Metrics measured for this include time for surgery, length of hospital stay, morbidity and mortality. However, the most commonly reported metric is operative time. Improving surgical intuition can reduce operative times, reduce average and peak forces, and improve accuracy and surgical success rates [81]. Creating a realistic feel for the operating surgeon, whose visual cues are now directed through an indirect and time‐delayed visual reconstruction and whose hands are no longer in contact with the patient, poses challenges.

#### 3.5.2 Challenges and Current Research

Challenges to the human operator's intuition include the lack of haptic feedback, mirroring and scaling effects, friction, and reduced DOF [7]. To offset the challenges of the mirroring and scaling effects, modern surgical robotic systems can provide motion scaling (the appropriate scaling and transformation of master movements to slave movements), motion filters, 3D and augmented vision, as well as compensated hand‐eye coordination [82]. The need to frequently clutch, that is, temporarily disconnect the connection between the surgeon‐side movement and the instrument movement to allow for repositioning, can create a cognitive and ergonomic burden for surgeons. Adaptive motion scaling has been shown to reduce the need for clutching and the associated cognitive burden [83]. Accurate instrument tracking creates an immersive experience and can be performed with vision‐based computational techniques utilising virtual markers [84], and safe surgical instrument trajectories can be created with guided virtual fixation pipelines [85]. Improved haptic technology can convey force and tactile sensations from the robot end effector via operator force, vibration, and skin stretch, and can be used to improve the realism of surgery [86]. Finally, challenges with communication, reliability, and fatigue of the bedside team can decrease surgical efficiency. Technology that improves communication with the bedside team and even reduces reliance on the bedside team, such as an extra arm that automates instrument exchange, may improve communication errors and operative time.

#### 3.5.3 Surgeon's Perspective

From a surgeon's standpoint, any additional technology that will reduce operating cognitive burden will increase surgeon productivity, technology usage, and reduce learning curves. As surgical robot use is expanded to more and more procedures, this will create a larger pool of providers who could benefit themselves and their patients from performing select cases with robot assistance. There is a large learning curve to acquiring the clinical expertise and obtaining and maintaining medical facility privileges with current robotic surgery systems. If a surgeon feels that a surgical case performed with versus without a robot will cost more, take longer, be less safe, and be more technically challenging due to the limitations discussed, they may find themselves using the robot less frequently and even give up maintenance of privileges. The pursuit of technological innovations that improve intuition, improve surgeon ergonomics, reduce operating time, and reduce complications will entice a wider community of providers in the use and trust of these systems.

## 4 Discussion and Future Directions

This review discusses fundamental elements of surgical robotic systems and current advances in the field. Each of these subsystems warrants an entire volume of discussion, and, thus, only a broad overview can be supplied. The ultimate purpose of a robotic surgical system is to function as a means to improve surgeon efficacy, efficiency, comfort and, most importantly, to improve patient outcomes. This section will be organised into a few imperatives for future research.

### 4.1 Improving Teleoperation Control, Security, and Long‐Distance Speed

An additional area of critical concern is cybersecurity. Robotic surgical systems have the ability to store critical information, including patient personal health information (PHI), patient videos, surgical data logs, and surgeon performance information. Interrupting a surgical robot in contact with a patient can cause major harm. There are multiple areas of vulnerability of these robots, including at the level of hospital infrastructure, system network, system updates, and intra‐operative real‐time internet connectivity [87]. Ensuring security of robotic surgical systems requires the vendors to have scrupulous attention to security with hardware and software design and requires hospitals to maintain a close relation with vendors to stay up to date with security updates and to maintain a general atmosphere of cybersecurity hygiene [87]. As technological capacity improves, there is an increasing opportunity to perform remote surgery in under‐served areas, which would pose additional security challenges. Current wireless teleoperation systems can suffer from time delays over long distances for which multiple control schemes have arisen to facilitate the sensation of telepresence. The main goals in this front are to improve data transfer, processing efficiency, and accuracy and optimise control schemes to reduce the undesired overshoot that can result from an overcompensated surgeon response to a delayed visual or force feedback.

### 4.2 Optimising Kinematics Computing Speed, Algorithms, and Accuracy

For kinematics and dynamics control, improving computer processing speed can improve existing algorithm calculations. Optimization of recursion algorithms may also allow for more efficient end effector motion calculations and trajectory generation. Machine learning from high volume trajectory generation and end effector and joint force calibration trained under both free motion and forced motion environments may improve end effector pose accuracy. Additionally, external force or positioning sensors or cameras may supplement existing methods of pose dynamics, and this has been demonstrated, for example, with flexible sensor sleeves overlying the joints of the robot or human operator [88].

### 4.3 Exploring Alternate Actuation Systems

The most common actuation system for minimally invasive endoscopic surgery uses tendon sheath actuation, in which an actuating wire is contained within a hollow core sheath [89]. This has advantages of high force transmission capability but is also challenged by friction, compliance, non‐linear transmission and tendon deformation [90]. Soft and continuum robots have emerged as an alternative system for minimally invasive surgery. For continuum robots, these can be extrinsically or intrinsically actuated. Extrinsic actuation systems include cable‐driven and concentric tube actuations. These can be limited by frictional damage to surrounding tissues, hysteresis, elastic instability, and limited load capacity. Intrinsic actuation mechanisms include pneumatic, hydraulic, shape memory alloy, and electroactive polymers. Intrinsic actuation mechanisms can have limited load capacity, thermal dissipation risk, excessive weight and size, and slow response time [91]. The use of these systems may extend the range of motion surgical instruments, which is particularly critical in the tight confines faced during robotic surgery.

### 4.4 Improving Endoscopic Surgical Visualisation

A major challenge with existing robotic surgery systems is increasing flexibility of the endoscopic camera and reducing the need to reposition and clean the camera. The endoscopic camera could be improved through further miniaturisation and flexibility. Given that the camera is positioned prior to instrument insertion, a potential solution to the issue of the endoscopic camera colliding and obstructing the operating instruments is through the use of continuum‐based actuation, a proximal torque sensor, and the creation of an RCM with the camera tip. In this manner, after the camera tip is positioned in an ideal location for the operating site, if an obstruction is created by camera shaft, the proximal torque caused by the operating instruments colliding against the endoscopic camera shaft could force the system to recalibrate the endoscopic camera shaft position while maintaining the RCM at the camera head. This would prevent the need to frequently reposition the camera and would allow for smoother surgery [92]. The addition of a self‐cleaning mechanism may also reduce the need for camera retraction and repositioning.

### 4.5 Providing Realistic, Real‐Time Haptic Feedback

A lack of accurate haptic feedback is cited as the most important limitation to current robotic surgical systems [93]. This topic is addressed in two related sections, end‐effector sensing and surgeon‐side haptic feedback.

#### 4.5.1 Developing Accurate, Resilient End‐Effector Sensors

For a robotic end‐effector sensor to serve as an extension of the surgeon fingertip when palpating the complex surface of human tissue, it is important to develop a convex detection platform that can be miniaturised sufficiently to pass through the operating trocar ports, which typically range in size from 3 to 15 mm, with the most commonly used sizes being 5 and 10 mm [94, 95]. It is known that a smaller access port can reduce post‐surgical pain [95, 96]. These considerations warrant optimising sensor size to be under 10 mm, and, ideally, under 5 mm, which is multiple fold smaller than the size of commercial miniature tactile sensors such as the GelSight Mini, which has a widest diameter of 32 mm [97]. Additional factors to consider are sensor resilience, the ability to maintain accuracy after a large volume of palpations, reusability for economic feasibility, and resistance to the high temperature and pressure environments of sterilisation. Piezoelectric materials possess great potential for the creation of smaller robotic end effector sensors, particularly with incorporation into micro‐electro‐mechanical systems (MEMS) arrays. However, it is important to create safer materials for biocompatibility, biodegradability and high performance without the bio‐toxicity of lead. Ceramic‐based flexible piezoelectrics are more brittle, while stretchable material‐based piezoelectrics have low performance [45]. Novel piezoelectric crystals, grain organisation, ceramic composition, surface depositions for thin films, and polarization techniques may allow optimization of the performance of flexible, biocompatible piezoelectric MEMS arrays. For optical sensors, improvements in light guide arrangement, light ray and camera geometry, data processing, and camera efficiency would be of benefit. Additionally, given the new progress in event cameras and the reduced data burden they pose during times of limited activity, the combination of RGB and event cameras may be of benefit [62]. Given the advanced complexity and sensitivity of both human and other vertebrate fingertips, including the ability to detect slip even on wet surfaces, bio‐inspired sensor designs have also been avidly pursued [98]. The human fingertip curvature allows greater propensity of signals obtained from force normal to skin ridges, which may increase sensitivity [99]. Bio‐mimetic designs incorporating these features may improve sensor sensitivity and allow further miniaturisation. Given the anisotropic and non‐linear nature of human tissue, when utilising machine learning techniques for calibrating sensor accuracy, it is important to present the challenge of both linear and non‐linear surface force and texture detection and static and dynamic tissue and off‐normal approach angles [100]. As previously discussed, the incorporation of evolving light‐, electrical‐, and stiffness‐based sensors into the robotic tool tip can assist with disease identification and treatment. Finally, while still in early stages of development, bio‐ and chemosensors, based on the principle of converting a chemical detection to an electrical signal, are being developed into chip platforms for point of care testing [101]. If these detection platforms are able to be converted into mobile, live sensing units, the electrical signal feedback could be used to inform surgical decision‐making and even guide robot movement.

#### 4.5.2 Integrating an Ergonomic, Stable Haptic Feedback Controller

Two of the most prominent surgical robots, DaVinci (Intuitive, Sunnyvale, CA) and Hugo (Medtronic, Macquarie Park, NSW) feature smaller hand held controls that predominantly interact with the surgeon fingertip. The utility of a larger surface area haptic interface may allow for more complex articulated surgical tools that feature more DOF for soft tissue manipulation and a wider surface area for haptic feedback. Exoskeletons have been demonstrated to be effective in robotic teleoperation and may represent the next frontier in robotic surgery, particularly if enhanced tool geometry and movement can be created with advances in soft and continuum robots [102]. These may additionally be used to register surgical movements for robotic learning, optimise surgeon ergonomics, and provide corrective haptic feedback if an erroneous surgeon trajectory is detected. The incorporation of a larger haptic interface such as a hand or hand‐forearm exoskeleton may reduce surgeon fatigue, allowing more natural movements, and allow a larger surface area for haptic feedback. Incorporation of finer detail haptic feedback features, such as stretch and pinch perception on the skin, may also enhance surgeon immersion. These can be integrated with non‐invasively applied topical magnets and the use of electrical and vibrational stimulation [103, 104].

### 4.6 Accurately Render Real‐Time Anatomy and Assist Surgical Movements

The ability to accurately render a 3D image of pre‐surgical anatomy, suspected abnormalities such as tumours, and the remodelled anatomy after certain surgical manipulations would greatly enhance a surgeon's intuition. Accurate end‐effector sensing and computer vision can play a role in this development. Robotic synesthesia involves collecting information from both visual and tactile point clouds to improve object estimation accuracy [105, 106, 107]. Incorporating a combination of surgeon‐driven or automated end‐effector palpations with vision‐ and tactile‐based sensors may allow for improved inclusion detection to guide accurate tumour mapping. Palpations could be used to geometrically reconstruct and display anatomy to a surgeon to guide accurate tumour margin resection and even to allow for autonomous surgical resection [108, 109, 110]. Automated robotic movements, with motion independent of the surgeon, can be beneficial in certain high‐precision tasks and reduce surgeon strain. Accurate end‐effector tactile sensors can be used to refine a policy, the mapping between sensor observations and robot actions, and allow automatic robotic movements [111]. Vision‐based learning models can be created and trained in simulation and can potentially be used to improve autonomous surgical incision, cannula insertion, and tumour tracking [112, 113, 114, 115, 116, 117, 118]. Augmented reality can be incorporated to improve surgeon situation awareness and improve instrumentation precision [119].

## Funding

The authors have nothing to report.

## Conflicts of Interest

Authors N.S., W.F., A.B., M.W.S., S.A., and M.A.A. all declare they have no financial interests. Author DS has received institutional research funding from the Intuitive Foundation (Sunnyvale, CA). Author M.T.T. has received honoraria and research grant funding from Intuitive Surgical Inc. (Sunnyvale, CA).

## Data Availability

Data sharing not applicable to this article as no datasets were generated or analysed during the current study.
